# Supracricoid hemilaryngopharyngectomy for selected pyriform sinus carcinoma patients – a retrospective chart review

**DOI:** 10.1186/1477-7819-7-65

**Published:** 2009-08-11

**Authors:** George X Papacharalampous, Georgios P Kotsis, Petros V Vlastarakos, Alexandros Georgolios, Ioannis Seggas, Ioannis E Yiotakis, Leonidas Manolopoulos

**Affiliations:** 1 A' ENT Department, Athens University, Medical School, 114 Vass. Sophias av. 11527 Athens, Greece; 2 ENT Department, Elpis General Hospital, 7 Dimitsanas St, 11528 Athens, Greece

## Abstract

**Background:**

The aim of this study is to assess the functional and oncologic results of supracricoid hemilaryngopharyngectomy and report our experience in the technique, local control and overall survival rates.

**Materials and methods:**

18 selected patients with pyriform sinus cancer treated by supracricoid hemilaryngopharyngectomy in a University Hospital setting. Retrospective chart review was used to assess functional and oncologic results of the procedure.

**Results:**

The actuarial 5 year survival rate in our study was 55.56% and the actuarial neck recurrence rate was 16.67%. All patients were successfully decannulated. Aspiration pneumonia was the most common postoperative complication (22.23%) and was treated mostly conservatively. One patient required a temporary gastrostomy but no patient needed total laryngectomy in the postoperative period.

**Conclusion:**

Supracricoid hemilaryngopharyngectomy in experienced hands is a reliable technique for selected patients with pyriform sinus cancer.

## Background

The pyriform sinus is the most common site of origin of hypopharyngeal cancer accounting for almost 70% of hypopharyngeal carcinoma cases (Pingree T.F. 1987)[1], followed by the posterior wall (20%) and the postcricoid region (Carpenter R.J. 3rd 1977)[2]. Surgery alone or with radiotherapy or chemotherapy is involved in the therapeutic strategy of almost 74% of pyriform sinus cancer patients in the USA (Hoffman H.T. 1997)[3]. Except for the earliest of lesions, total laryngopharyngectomy is the surgical treatment of choice, whereas neck dissection is generally performed if there is a N1-N3 palpable adenopathy or clinical N0 neck but a T3-T4 primary tumor (Teknos T.N. 2001)[4].

**Figure 1:**
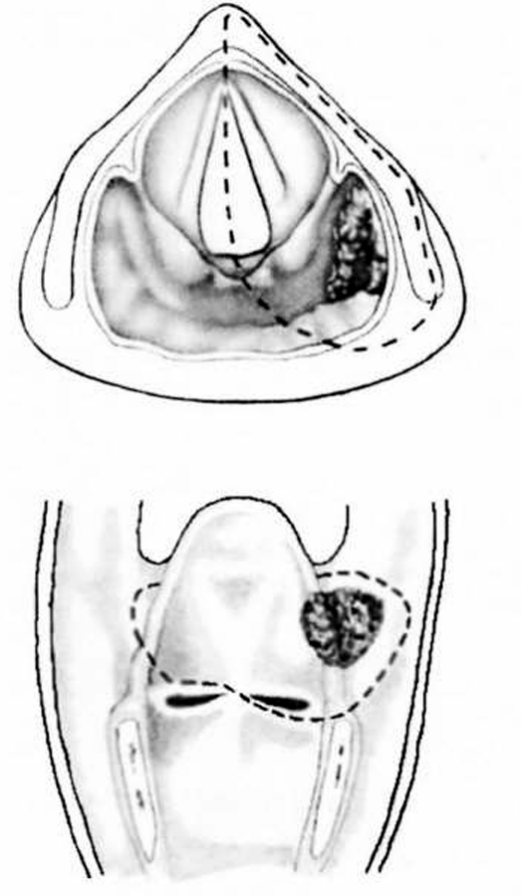
**Supracricoid hemilaryngopharyngectomy: representation of the resection for a pyriform sinus neoplasm**.

The supracricoid hemilaryngopharyngectomy was first introduced in 1965 (Andre P. 1965)[5] and it is indicated for selected cases of malignancies located in the pyriform sinus and the lateral wall of the larynx (Figure 1). The procedure consists of removal of the supracricoid hemilarynx and ipsilateral pyriform sinus.

We retrospectively reviewed selected cases of patients with pyriform sinus carcinoma treated by supracricoid vertical hemilaryngopharyngectomy in our service between 1994 and 2002. The objective of our study is to assess the functional and oncologic results of the procedure and report our local control and overall survival rates.

## Materials and methods

From 1994 to 2002, 18 selected patients with pyriform sinus cancer were treated in the Department of Otorhinolaryngology Head and Neck Surgery, Hippocrateion Hospital, University of Athens Medical School. All patients were treated by supracricoid hemilaryngopharyngectomy and unilateral or bilateral neck dissection. The patients were followed postoperatively at least for 5 years or until their death. Our exclusion criteria were: a) Other histologic type than squamous cell carcinoma; b) extension of the neoplasm to the pyriform sinus apex, the pre-epiglottic or para-epiglottic space, the thyroid cartilage or endolarynx, the base of the tongue and posterior tonsillar pillar, the posterior pharyngeal wall and the postcricoid region.

All patients were male and the mean age was 55.7 (range 44 to 70). Ten patients were staged as T2N0M0, six patients were staged as T2N1M0, one was staged as T2N2M0 and one patient was staged as T3N0M0. Extensions of the tumor from the pyriform sinus to the adjacent structures are presented in Table 1. All patients were smokers (Table 2). Nine out of the 15 patients that were considered as heavy smokers, admitted heavy alcohol consumption in a regular basis, too.

**Table 1:** Involvement of adjacent sites (except pyriform sinus).

Adjacent site involved	Number of patients	Percentage of patients
Aryteno- epiglottic fold	3	16.6
Arytenoid cartilage	12	66.6
Vallecula	1	5.5
False vocal cord	2	11.1

**Table 2:** Smoking.

Pack-years	1–20	20–40	40–60	>60
Number of patients	3 (17%)	8 (44%)	5 (28.5%)	2 (11.5%)

The patients were assessed initially by panendoscopy under general anesthesia and bioptic material was taken to confirm the malignant potential and histologic type of the neoplasm. Preoperatively, patients underwent complete blood count, basic metabolic and coagulation profile panels and the routine cardiologic evaluation. Computed tomography and barium studies were included in the preoperative evaluation and tumor staging, as well.

The postoperative care was standardized for all patients. A low pressure cuffed tracheostomy cannula was maintained at least until the third postoperative day. The decisions for removal of the nasogastric tube and initiation of oral feeding were taken in the lack of aspiration episodes indicating adequate swallowing mechanism. Furthermore, we offered gastrostomy to the patients that had not achieved a satisfying swallowing function by the end of the 4^th ^postoperative week. Finally, all patients with pathologically confirmed nodal disease, extracapsular spread or positive surgical margins received postoperative radiation. Laryngeal shielding and "small size field" techniques were involved in all radiated patients in order to minimize post radiation laryngeal edema and preserve laryngeal function.

## Results

### Functional Results

No patients died in the postoperative period. Two patients (11.1%) developed hematoma that did not need surgical intervention. Wound infection occurred in one patient (5.5%) and did not require further surgery. Aspiration pneumonia that was confirmed by radiologic imaging, occurred in 4 patients (22.2%) and was treated with antibiotics and chest therapy. One patient presented multiple aspiration pneumonia episodes and was finally submitted to gastrostomy 40 days after the surgical operation.

All patients were decannulated. The average time until decannulation was 7 days (range 3–97 days). Decannulation was delayed beyond the third day only in 5 out of 18 patients. The average time until the removal of the nasogastric tube was 20 days. In 12 patients (67%) the NG tube was removed before the 11^th ^postoperative day. In 2 patients, diagnosed with aspiration pneumonia, the NG tube was removed the 28^th ^and 29^th ^postoperative day, respectively. As mentioned above, one patient underwent gastrostomy 40 days after the surgical operation.

### Oncologic results- Survival

#### 3-years postoperative results

The average follow-up time was 88.2 months (range 70–108 months). The 3 year actuarial survival rate was 77.78% (14 out of 18 patients). Local recurrence occurred in one patient in the first 3 year period (5.56%). Neck recurrence occurred in two patients (11.12%) and two patients presented with metachronous second primary site in the head and neck (11.12%). Totally, 4 patients died in the 3 year postoperative period, two patients as a result of second primary site tumors and two succumbing to distant metastatic disease.

#### 5 year postoperative results

**Figure 2:**
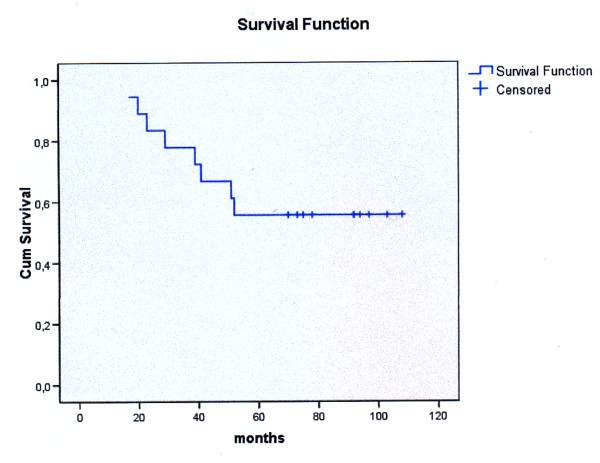
**Overall 5-year survival rate, Kaplan-Meier analysis**.

The actuarial 5 year survival rate in our study was 55.56% (10 out of 18 patients). (Figure 2). Local recurrence rate was as in the first 3 year period (5.56%), as no patient appeared with recurrences after the 3^rd ^year. Overall, neck recurrence occurred in three patients (16.67%) as there was one more patient diagnosed with neck disease after the 3^rd ^year of his follow-up. After the 3 year period, there were no more patients diagnosed with distant metastatic disease, but two more patients were diagnosed with a second primary malignancy, making an overall rate of 22.23% (4 patients) for the 5 year period. In Table 3 we present the overall death rate for the 5 year postoperative period and the relevant cause of deaths.

**Table 3:** Etiology of death, 5 year period post-operation.

Etiology of death	Number of patients	Percentage
Local recurrence	0	0
Neck recurrence	1	5.56%
Distant metastasis	2	11.12%
Second primary site	4	22.23%
Other	1	5.56%

## Discussion

Pyriform sinus carcinoma has a 5-year disease specific survival of 33.6% (Gourin C.G. 2004)[6]. In our series the 5 year survival rate was 55.56%, which can be attributed to the relatively low number of cases, since the technique and postoperative treatment was according to the standard of care. All patients were treated by supracricoid hemilaryngopharyngectomy and unilateral or bilateral neck dissection. Resection margins were assessed by frozen sections intra-operatively and were free of tumor in all cases. The technique was used as previously reported in the literature (Laccourreye H. 1987[7]; Laccourreye O. 2005[8]) and the strategy regarding the neck treatment was individualised for every patient but consistent with previous reports in the literature (Kania R. 2005)[9]. Therefore, the N0 patients were treated by unilateral modified radical neck dissection (levels I-V were removed, sternocleidomastoid muscle, internal jugular vein and spinal accessory nerve were preserved) and the N1/N2 patients underwent unilateral radical neck dissection (levels I-V). Out of the 10 patients with clinically negative neck, 2 were identified to have nodal disease in the surgical pathology report (one had extracapsular spread and one multiple nodal involvement (Table 4). We performed homolateral radical neck dissection (levels I-V) and contralateral selective neck dissection (levels II, III, IV) to the T3N2M0 patient, in which the tumor was identified to cross the midline in the supraglottis. In all patients we performed thyroid isthmectomy and unilateral thyroid lobectomy in the side of the lesion.

**Table 4:** Comparison of clinical and pathological staging*

Clinical staging	(-)	(+)	(+), ECS	(+) >1 nodes involved	(+), ECS >1 nodes involved
N0	8	0	1	1	0
N1	2	0	1	2	1
N2	0	0	1	0	1

(-): negative pathology, (+): positive pathology, ECS: extracapsular spread

Aspiration is well established as the main risk following conservative surgery in the hypopharynx (Krespi Y.P. 1985[10]; Krespi Y.P. 1984[11]; Yoo S.J. 2000[12]). This complication is the result of sacrificing the superior laryngeal nerve (Teymoortash A. 2007[13]) in head and neck surgery (Finck C. 2006)[14], whereas incidences of permanent gastrostomy, completion total laryngectomy, and aspiration-related death have been reported to 0.7%, 1.5%, and 0.7%, respectively in relevant studies (Laccourreye O. 2005)[8]. In our series, aspiration pneumonia was diagnosed in 4 patients (22.2%) and was treated conservatively. One patient presented with recurrent episodes and was finally submitted to gastrostomy 40 days after operation. After this intervention, the patient did remarkably well and was able to receive per os feeding by the end of the second postoperative month. No patient in our series needed total laryngectomy or other neck surgery for the management of permanent aspiration.

A second primary tumor was encountered in 22.23% (4 out of 18 patients) of our patients in the 5 year postoperative follow-up period. The second primary tumor was the main oncological cause of death in our cohort. Continued smoking and alcohol consumption by our patients postoperatively can explain the appearance of these metachronous lesions. In head and neck cancer, the probability of developing a second metachronous cancer 5-years after undergoing treatment for the initial tumor is 22% and the second malignancy is almost always fatal (Schwartz L.H. 1994)[15]. Distant metastasis was diagnosed in 11.12% (2 out of 18 patients), close to previous reports (Marks J. E. 1978)[16]. In both cases the disease was lethal and identified in the first 3 years of the study. The lymph node disease at the time of operation was 55.5% (8 out of 18 patients had negative pathology reports), similar to the 59.4% and 70.8% lymph node metastasis rates that have been reported for patients T2 and T3 pyriform sinus disease, respectively (Shen N. 2007). The 5 years postoperative cervical node recurrence was 16.67% (3 out of 18 patients) and was fatal for one patient.

## Conclusion

Supracricoid hemilaryngopharyngectomy is a reliable technique for selected patients suffering from pyriform sinus carcinoma. The main postoperative complication is aspiration pneumonia which is commonly amenable to conservative measures. Nevertheless, a total laryngectomy or other surgical intervention for the management of permanent aspiration is not a common event. Distant metastasis, neck recurrence and second primary tumor are major concerns for the surgeon in the postoperative follow-up period of these patients.

## Competing interests

The authors declare that they have no competing interests.

## Authors' contributions

GP participated in the design of the study, performed the statistical analysis and drafted the manuscript. All other authors conceived of the study, and participated in its design and coordination. All authors read and approved the final manuscript.
